# Maternal Psychological Control and Rural Left-Behind Children's Anxiety: The Moderating Role of Externalizing Problem Behavior and Teacher Support

**DOI:** 10.3389/fpsyg.2021.624372

**Published:** 2021-07-05

**Authors:** Na Deng, Hongyan Bi, Jinxia Zhao

**Affiliations:** ^1^Institute of Psychology, Chinese Academy of Sciences, Beijing, China; ^2^College of Education, Linyi University, Linyi, China

**Keywords:** psychological control, externalizing problem behaviors, teacher support, anxiety, left-behind children, China

## Abstract

Based on the risk and protective factor framework and context-dependent theory, the present study investigated the association between maternal psychological control and anxiety among left-behind children in rural China as well as the moderating roles of externalizing problem behavior and teacher support in this association. A total of 149 children with two migrant parents, 306 children with only a migrant father, and 287 accompanied children aged 11–16 years (M = 13.85 years, SD = 0.50) in the rural areas of Shandong Province, Eastern China, participated in the study. Children reported their perceived levels of maternal psychological control, teacher support, externalizing problem behavior, and anxiety. The results provided evidence that a higher level of psychological control was associated with more anxiety symptoms in all three groups of children, and this association was stronger in children with two migrant parents than in accompanied children. Hierarchical regression analysis supported our hypotheses that the moderating effects of externalizing problem behavior and teacher support varied as a function of parents' migrant status, with externalizing problem behavior exacerbating the impact of psychological control on anxiety in accompanied children, and teacher support buffering the impact of psychological control on anxiety in children with a migrant father. These findings broaden our understanding of the factors and processes that contribute to anxiety problems in left-behind children in rural China.

## Introduction

Anxiety, generally defined as a negative emotional state of vigilant apprehension characterized by a narrowing of attention, a sense of dread, ruminative worry, a perception of vulnerability, an inhibition of behavior, and a heightened state of sympathetic arousal (American Psychiatric Association, 2000), is one of the most prevalent emotional disorders in childhood, with prevalence rates of approximately 5–13% in community children younger than 18 years old (Moreno et al., 2010) and 26.6% in rural children and adolescents aged 3–19 years (Abbo et al., 2013). Childhood anxiety disorders are associated with poor academic performance, depression, increased risk for substance abuse, and other externalizing disorders (Whiteside and Brown, 2008; Kendall et al., 2010). If left untreated, such disorders may persist into adolescence and even adulthood (Broeren and Muris, 2008). All these findings highlight the importance of identifying factors that contribute to anxiety problems in childhood. In rural China, labor migration from rural to urban areas has been the most significant social change since the reform and opening policy was implemented in 1978. With labor migration, a large number of children aged 17 years or younger have been left behind in the original rural communities by one or both parent migrant in search of work in cities, which has led to dramatic changes in the traditional family structure (Cheng and Sun, 2014). Parent-migrant/other caregiver families and father-migrant/mother caregiver families have increasingly become two common patterns of migrant families in rural China (Zhao et al., 2015). Although migrant workers provide more economic resources for their children, most of the left-behind children actually live in a situation lacking parental or paternal care and nurture, which might lead to an increase in anxiety symptoms. Indeed, empirical studies (Li and Cai, 2012; Cheng and Sun, 2014) have found that left-behind children reported higher levels of anxiety than their age-matched peers from non-migrant families. However, of note, most of the prior research did not distinguish between types of migrant families. Extant research has demonstrated that family structures affect parenting and family communication patterns and, subsequently, affect children's adjustment (Bastaits and Mortelmans, 2016; Murry and Lippold, 2018). To the best of our knowledge, the only study that has examined the differences in children's levels of anxiety between types of migrant families found that children with two migrant parents displayed higher levels of anxiety than those with only a migrant father (Zhao and Li, 2017). Thus, one might wonder whether children with only a migrant father might display higher levels of anxiety than accompanied children in non-migrant families, considering that the loss of contact with fathers is an important risk factor for and has been shown to be associated with maladjustment among children (Carlson, 2006). Based on these findings, the present study examined differences in anxiety levels among two-migrant-parent children, only-a-migrant-father children, and accompanied children.

### The Relationship Between Parental Psychological Control and Child Anxiety

Research on the etiology of anxiety often begins with an examination of parenting, due to the importance of the family microsystem in children's development. Control is considered one of the most influential dimensions of parenting. Prior research has focused on two types of control—psychological and behavioral—and documented their distinct effects on children's functioning (Pomerantz and Wang, 2009; Bebes et al., 2015). In contrast to behavioral control—i.e., control over children's activities and behaviors in the physical world, which benefits children's academic and behavioral adjustment (Wang et al., 2007)—a large body of research has shown that psychological control—i.e., control over children's thoughts and feelings in the psychological world, which intrudes upon children's sense of self (Wang et al., 2007)—is closely associated with children's anxiety (Bebes et al., 2015; Stone et al., 2015; Zhao et al., 2018). Nevertheless, almost all of the studies in this area have been conducted in children of intact families; there is a lack of research on the association between psychological control and anxiety of left-behind children from families of different parental migrant statuses. Murry and Lippold (2018) proposed that changes in family structure, as a consequence of parental migration, affect the levels of parenting, including parental involvement, control, and warmth, which are likely to influence children's adjustment and well-being. Context-dependent theory suggests that chronic and cumulative stress (i.e., parental absence caused by different forms of family separation such as divorce, death of a parent, separation, and migration) may increase an individual's reactivity (i.e., behavioral or emotional arousal), and highly reactive individuals may be more susceptible to both negative (risk-promoting) and positive (development-promoting) environments (Ellis and Boyce, 2011; Steeger et al., 2017). Empirical support for this viewpoint has come from several studies on the associations between parenting or parent-child relation and children's well-being in diverse family structures. Specifically, a study by Bloch et al. (2007) found that children growing up in a household in which the parent is absent due to death or divorce are more prone to exposure to stressful life events than those in intact families. Zhao and Li (2017) found that the relationship between insecure attachment with fathers and adolescents' anxiety was stronger in families with two migrant parents (*r* = 0.21) than in families with only a migrant father (*r* = 0.13). Li and Liu (2013) reported that a high level of secure attachment with fathers directly increased subjective well-being among children with one or both migrant parents (*ß* = 0.34) but not among accompanied children (*ß* = 0.06). Based on these indirect evidences, the present study further examined the difference in the association between perceived psychological control parenting and children's anxiety among two-migrant-parent families, only-a-migrant-father families and non-migrant families to test whether the extent of the association between psychological control and children's anxiety depended on parents' migrant status.

### The Moderating Effect of Externalizing Problem Behavior

Although higher levels of parental psychological control are associated with higher levels of child anxiety, some research has found that psychological control is not uniquely related to child anxiety (Caron et al., 2006; Gere et al., 2012), which to some extent suggests that the significant association between psychological control parenting and children's anxiety reported in prior research (Stone et al., 2015; Zhao et al., 2018) may be influenced by other potential confounding variables. Externalizing problem behavior refers to behavior problems that are manifested in children's outward behavior and reflect children's negative actions in the external environment, such as aggression and delinquency (Campbell et al., 2000). It has been documented that parental psychological control is related to children's externalizing problem behavior (Symeou and Georgiou, 2017; Pace et al., 2018). Given that children with anxiety disorders often have cooccurring externalizing behavior disorders (Kendall et al., 2010), it is therefore crucial to consider behavioral problems when examining the relationship between parental psychological control and children's anxiety. To date, only a few studies have examined the role of child externalizing problem behavior in the association between parenting and children's anxiety or internalizing symptoms. In studies of community children from primary schools, it has been reported that the significant associations between psychological control (Caron et al., 2006) or parenting patterns (Pereira et al., 2009), and children's internalizing symptoms disappeared after controlling for cooccurring externalizing symptoms. Similarly, in a clinical sample of 190 children aged 7–13 years, Gere et al. (2012) found that the previous significant relationship between parental overprotection, often referred to as parental control, and children's anxiety became non-significant after controlling for children's externalizing symptoms. To some extent, these findings demonstrated that externalizing problem behavior may moderate the association between psychological control parenting and children's anxiety; that is, this association may be significant at a higher level of externalizing behavior, but it may become non-significant at a lower level of externalizing behavior. However, these studies have not specifically focused on the question of whether the role of externalizing problem behavior in the association between parental psychological control and children's anxiety changes across different family structures. In two-migrant-parent and only-a-migrant-father families in rural China, Wen and Lin (2012) argued that, irrespective of the level of other sources, parental or paternal absence has considerable emotional costs for left-behind children's development due to weakened parental support and guidance, and undermined parent–child bonding. Additionally, according to the opinion of context-dependent theory, long-term absence of parental or paternal support in migrant families may be an important source for children's high reactivity to negative environments that lead to maladaptive outcomes (Obradović et al., 2010; Steeger et al., 2017) regardless of the level of other risk factors. Considering left-behind children's high reactivity to negative environments, one might wonder whether psychological control parenting was always associated with left-behind children's anxiety regardless of the level of their externalizing problem behavior. In other words, one might wonder whether the absence of both parents or fathers make the influences of externalizing problem behavior on the association between psychological control and left-behind children's anxiety decrease or disappear. Therefore, the third aim of this study was to examine how the moderating effects of externalizing problem behavior on the association between psychological control and children's anxiety differed among two-migrant-parent, only-a-migrant-father, and non-migrant families.

### The Moderating Effect of Teacher Support

To date, most research on anxiety has focused on the risk factors for family microsystems (e.g., negative parenting, parental anxiety; Eley et al., 2015; Möller et al., 2015) and individual characteristics (e.g., behavioral inhibition; Murray et al., 2008). There is a lack of research on protective buffers against the negative impact of risk situations on child anxiety. According to the risk and protective factor framework, child development is the dynamic interplay between risk and protective factors whereby risk factors predispose children to negative developmental outcomes, and protective factors increase resilience and decrease the likelihood of negative outcomes (Masten, 2001; Wang et al., 2013). School is another important microsystem factor for child development, and teachers are the primary adult figures within this microsystem. Teacher support, often defined as “children's perceived or actual instrumental and/or expressive provisions supplied by the teacher” (Lin, 1986), as a direct, immediate, or proximal contextual factor, may act as a protective factor between risk situations (e.g., psychological control) and children's anxiety. Indeed, numerous studies on child resilience have provided evidence to suggest that teacher support or high-quality teacher–student relationships serve as a buffer in the association between negative parenting and child psychological adjustment. For instance, Wang et al. (2013) found that positive experiences with teachers buffered adolescents with negative family experiences from engaging in misconduct behaviors. A study of maltreated children by Lynch and Cicchetti (1997) suggested that positive relationships with teachers compensated for negative relationships with parents. In addition, a study by Costa et al. (2005) suggests that positive relationship with teachers was particularly beneficial to students who do not have secure relationships with their parents. However, these studies mainly focused on children from intact families, and few studies have examined how the moderating effects of teacher support on the association between psychological control and anxiety differ among children left behind by both parents or children left behind by fathers only in rural areas. Because the potential therapeutic functions of social support in the context of adversity were underscored in the theory of interpersonal relationships by Sullivan (1953), when compared to accompanied children, teacher support may have a stronger protective effect against negative parenting on left-behind children's adjustment. Thus, the last aim of this study was to compare the moderating effect of teacher support on the association between parental psychological control and children's anxiety among families with different parents' migrant statuses.

### The Current Study

To extend our understanding of the risk and protective factors for children's anxiety beyond the results of previous research mostly conducted in intact families, the present study investigated the association between psychological control and children's anxiety in families with different parent migrant statuses in rural China as well as potential moderating mechanisms. Because psychological control was more likely to be used by parents as a means to make their children emotionally and psychologically dependent on them as children enter middle school—a time during which children's separation and independence increase (Soenens et al., 2010)—we focused on children who were at this stage of development. Given that mothers tend to be more involved than fathers in children's parenting (Wen and Lin, 2012), this study primarily focused on maternal psychological control. Based on the extant direct and indirect evidence, first, we expected that children with two migrant parents would report the highest levels of anxiety, followed by those with a migrant father and then accompanied children. Second, it was expected that maternal psychological control would be most closely associated with the increase of children's anxiety in two-parent-migrant families, followed by those in only-a-migrant-father families and then non-migrant families. Given the relative paucity of previous research on the moderating role of externalizing problem behavior in the association between psychological control and children's anxiety, we did not propose strong hypotheses on this issue. Finally, we anticipated that teacher support would be more likely to buffer the impact of maternal psychological control on left-behind children's anxiety (especially for the two-migrant-parent children) than that of accompanied children.

## Materials and Methods

### Participants

A total of 771 Chinese children aged 11–16 years enrolled in grade 7 were recruited from three junior high schools in three counties of Linyi, located in Shandong Province of Eastern China, which is a region that has a large labor migration population. According to the criterion employed by Zhao et al. (2012), a threshold of < 90% response rate was used to remove unsatisfactory cases. Twenty-nine students were excluded from the initial 771 participants. The final sample therefore consisted of 742 children, including 149 children in families with two migrant parents (M = 13.91 years, SD = 0.57; 100 boys and 49 girls), 306 children in families with only a migrant father (M = 13.84 years, SD = 0.43; 154 boys and 152 girls), and 287 accompanied children in non-migrant families (M = 13.83 years, SD = 0.52; 153 boys and 134 girls). Among children with two migrant parents, 85.7% of fathers and 96.7% of mothers had an educational level of junior high school or below, and the others had educational levels ranging from senior high school to junior college. Among children with only a migrant father, 87.3% of fathers and 98.1% of mothers had an educational level of junior high school or below, and the others had an educational level of senior high school or high. Among accompanied children, 84.7% of fathers and 94.3% of mothers had an educational level of junior high school or below, and the others had educational levels ranging from senior high school to junior college. Most of the fathers were unskilled workers (e.g., building workers, stevedores), and most mothers were farmers. The percentage of families in income per month per capita exceeding ¥1,000 was 71.6, 65, and 67.6% for families with two migrant parents, families with a migrant father, and non-migrant families, respectively.

### Procedure

We sought the help of the elementary school institutions for access to the participants of this study. Prior to data collection, approval to administer questionnaires was obtained from the school principals, and the students were assured of the voluntary and confidential nature of this research. The questionnaires were administrated to groups of students by a trained research assistant in separate classrooms. Students independently completed the Psychological Control Scale (Wang et al., 2007), Youth Self-Report List (Achenbach, 1991), Teacher (Significant Other) Support Subscale of the Multidimensional Scale of Perceived Social Support (MSPSS; Zimet et al., 1990), and Spence Children's Anxiety Scale (Zhao et al., 2012) during classroom hours or immediately after school. All procedures were approved by the Institutional Review Board at the Institute of Psychology, Chinese Academy of Sciences.

### Measures

#### Psychological Control

Maternal psychological control was assessed with an 18-item Chinese version developed by Wang et al. (2007). Ten items assessed *guilt induction* (e.g., “My mother tells me of all the sacrifices she has made for me.”), five assessed *love withdrawal* (e.g., “My mother avoids looking at me when I have disappointed her.”), and three assessed *authority assertion* (e.g., “My mother says, when I grow up, I will appreciate all the decisions she make for me.”). Child indicated how true each item was of his or her mother (1 = not at all true; 5 = very true). The mean score of the 18 items was taken, with higher scores indicating greater psychological control. The scale has been shown to have acceptable reliability and validity in Chinese middle school students (Wang et al., 2007). In the current sample, the alpha coefficient was 0.89 for the scale.

#### Externalizing Problem Behavior

Children's externalizing problem behavior was assessed with the Youth Self-Report List for ages 11–18 (YSR/11–18; Achenbach, 1991). The 30-item YSR externalizing scale comprises two subscales (aggressive behavior and delinquent behavior). Children rated each item (e.g., “I get into many fights.”) on a 3-point scale ranging from 0 (*not true*) to 2 (*very true or often true*). The responses for the 30 items were averaged, with higher scores indicating greater externalizing problem behavior. The Chinese version of the YSR has already been validated in Chinese children and adolescents and demonstrated adequate reliability and validity (Gershoff et al., 2010; Xing et al., 2011). The internal reliability of the scale was 0.87 in the current sample.

#### Teacher Support

The teacher (Significant Other) Support Subscale of the MSPSS (Zimet et al., 1990) was used to assess the children's perception of support from their teachers. On a scale ranging from 1 (never) to 5 (always), children completed four items, such as “There is a teacher who is around when I am in need.” The mean of the four items was taken, with a higher score representing greater teacher support. As a commonly used instrument for assessing children's perception of social support, the Teacher Support Subscale of the MSPSS has been validated in China and demonstrated adequate reliability and validity (Zhao et al., 2018). The alpha coefficient of the subscale was 0.81 in the current sample.

#### Children's Anxiety

The Chinese version of the Spence Children's Anxiety Scale (SCAS; Zhao et al., 2012) was employed to assess children's anxiety. The SCAS contains 44 items (e.g., “I worry about being away from my parents”), 38 of which can be grouped into six subscales: separation anxiety disorder (6 items), social phobia (6 items), physical injury fears (5 items), panic disorder and agoraphobia (9 items), obsessive-compulsive disorder (6 items), and generalized anxiety disorder (6 items). The remaining six statements are positively worded filler items. Children rated how often they experienced each symptom on a 4-point scale ranging from 0 (never) to 3 (always). The mean was taken, with higher scores indicating greater anxiety. The alpha coefficient of the scale was 0.93 in the present study.

## Results

### Descriptive Statistics and Correlations

A multivariate analysis of variance (MANOVA) was conducted to examine the group differences of the key variables (i.e., psychological control, externalizing problem behavior, teacher support, and anxiety), with parents' migrant status and children's gender as the between-group factors. The results of the MANOVA indicated that the combined dependent variables were significantly affected by children's gender [*F*_(4,733)_ = 24.91, *p* < 0.001, *η*^2^ = 0.12] and parental migrant status [*F*_(4,734)_ = 2.97, *p* < 0.05, *η*^2^ = 0.02]. As shown in Table 1, compared with girls, boys reported higher scores on maternal psychological control and externalizing problem behavior and lower scores on anxiety. *Post-hoc* tests revealed that, compared with accompanied children and children with a migrant father, children with two migrant parents had higher scores on psychological control and anxiety, and no significant difference in the two variables was found between children with a migrant father and accompanied children. The interaction between parents' migrant status and gender was not significant [*F*_(4,734)_ = 1.92, *p* > 0.05, *η*^2^ = 0.01]. Given that children's gender was associated with most variables of the current study, it was therefore included in subsequent analyses as a control variable.

**Table 1:** Comparisons of the key variables between parents' migrant status groups/children's gender groups (mean ± standard deviation).

	**Total by parents' migrant status**				**Total by children's gender**		
	**Cmp**	**Cmf**	**AC**	**Partial *η*^**2**^**	**Boys**	**Girls**	**Cohen's *d***
Psychological control	2.15 ± 0.58	2.03 ± 0.52	2.04 ± 0.55	0.01	2.14 ± 0.56	1.99 ± 0.52	0.28
Externalizing behavior	0.28 ± 0.24	0.25 ± 0.21	0.25 ± 0.19	0.00	0.28 ± 0.22	0.22 ± 0.18	0.30
Teacher support	1.19 ± 0.62	1.20 ± 0.64	1.19 ± 0.61	0.00	1.21 ± 0.65	1.18 ± 0.60	0.05
Anxiety	0.96 ± 0.54	0.87 ± 0.46	0.85 ± 0.48	0.01	0.83 ± 0.42	1.02 ± 0.49	−0.42

*Cmp, children with two migrant parents; Cmf, children with only a migrant father; AC, accompanied children*.

The partial correlations among all the key variables separated by parent migrant status and adjusted for children's gender are reported in Table 2. As shown, for the three groups, maternal psychological control was positively associated with children's anxiety. The online resource provided by Lowry (1998) was used to test the significance of the difference between two independent correlation coefficients. It was found that this association was stronger for children with two migrant parents than for accompanied children (*Z* = 2.36, *p* < 0.05). No significant differences in this association were found between children with two migrant parents and those with only a migrant father (*Z* = 1.52, *p* > 0.05) or between children with a migrant father and accompanied children (*Z* = 1.05, *p* > 0.05). Externalizing problem behavior was negatively associated with children's anxiety among the three groups. Teacher support was negatively associated with anxiety in children with two migrant parents and accompanied children but not in children with only a migrant father.

**Table 2:** Correlations among variables separately for two-migrant-parent children, only-a migrant-father children, and accompanied children.

	**1**	**2**	**3**	**4**
1. Psychological control	–	0.36^***^	0.01	0.24^***^
2. Externalizing behavior	0.35^***^ (0.35^***^)	–	−0.16^**^	0.43^**^
3. Teacher support	0.10 (−0.03)	−0.09 (−0.15)	–	−0.22^***^
4. Anxiety	0.32^***^ (0.45^***^)	0.43^**^ (0.54^***^)	−0.05 (−0.22^**^)	–

*Coefficients in parentheses below the diagonal are the correlations between variables for children with two migrant parents, and coefficients outside parentheses refer to children with a migrant father. Coefficients above the diagonal are the correlations between variables for accompanied children*.

** *p < 0.01;*

*** *p < 0.001*.

### Hierarchical Regression Analyses

To test whether the relationship between psychological control and children's anxiety was moderated by children's externalizing problem behavior and teacher support, and whether the specific moderating mechanisms depended on parents' migrant status, we conducted six hierarchical regression analyses. Regression analyses were performed separately for each type of family and each moderator. Gender was entered in the first step as a controlling variable, and the main effects of psychological control and the moderator were entered in the second step. The interaction term (psychological control × moderator) was entered in the third step. The online resource provided by Preacher et al. (2003) was used to estimate the simple slope. The values at 1 SD above and below the mean of the moderators were used to calculate the simple slopes of the association between psychological control and children's anxiety. Prior to conducting the regression analyses, all predictors were mean centered to reduce multicollinearity. Table 3 shows the results of six regression analyses.

**Table 3:** Summary of hierarchical regression analyses for the relationship between psychological control and anxiety with externalizing problem behavior/teacher support included as a moderator.

**Variables**	**Children with 2 migrant parents**		**Children with a migrant father**		**Accompanied children**	
	**β**	**SE**	***β***	**SE**	***β***	**SE**
**EPB as a moderator**						
Step 1						
Gender	0.23^**^	0.09	0.16^**^	0.05	0.24^***^	0.05
Step 2						
Gender	0.32^***^	0.07	0.24^***^	0.05	0.28^***^	0.05
PC	0.29^***^	0.06	0.19^***^	0.05	0.26^*^	0.05
EPB	0.43^***^	0.08	0.36^***^	0.07	0.38^***^	0.07
Step 3						
Gender	0.31^***^	0.07	0.25^***^	0.05	0.28^***^	0.05
PC	0.31^***^	0.06	0.19^***^	0.05	0.26^***^	0.05
EPB	0.50^***^	0.09	0.39^***^	0.09	0.36^***^	0.07
PC × EPB	0.12	0.11	0.07	0.10	0.13^*^	0.09
**TS as a moderator**						
Step 1						
Gender	0.23^**^	0.09	0.16^**^	0.05	0.24^***^	0.05
Step 2						
Gender	0.24^**^	0.07	0.18^**^	0.05	0.28^***^	0.05
PC	0.43^***^	0.06	0.33^***^	0.05	0.24^***^	0.05
TS	−0.20^**^	0.06	−0.09	0.04	−0.22^***^	0.04
Step 3						
Gender	0.24^**^	0.07	0.19^**^	0.05	0.28^***^	0.05
PC	0.44^***^	0.06	0.33^***^	0.05	0.24^***^	0.05
TS	−0.21^**^	0.06	−0.08	0.04	−0.21^**^	0.04
PC × TS	−0.07	0.09	−0.13^*^	0.06	−0.05	0.07

*PC, psychological control; EPB, externalizing problem behavior; TS, teacher support. Gender was coded 0 for boys and 1 for girls*.

* *p < 0.05;*

** *p < 0.01;*

*** *p < 0.001*.

**Figure 1:**
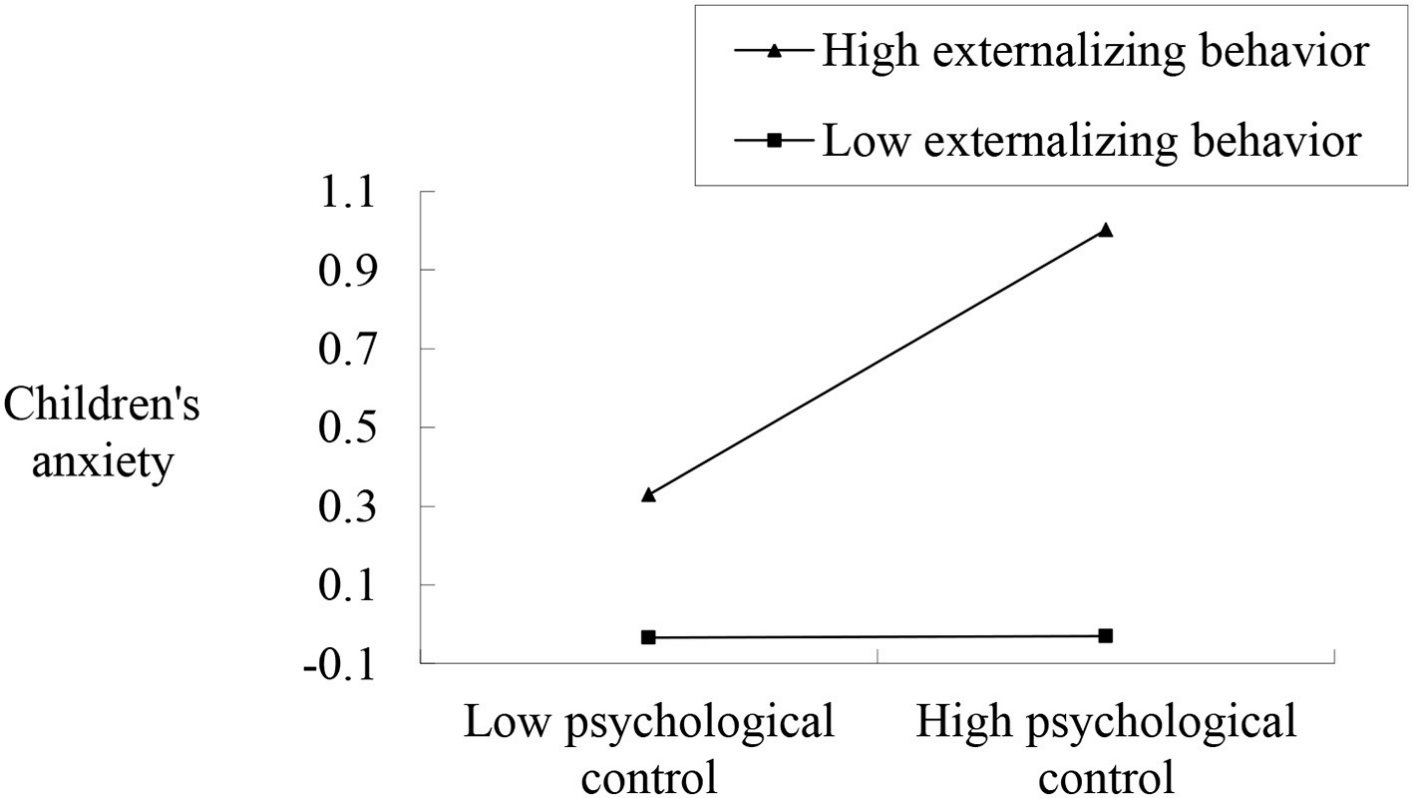
Interaction between maternal psychological control and externalizing problem behavior in predicting accompanied children's anxiety.

Three regression models testing the moderating role of externalizing problem behavior were computed first. The results showed that the regression models were all significant, explaining 40, 23, and 29% of the variance in anxiety for children with two migrant parents, children with a migrant father and accompanied children, respectively. Specifically, both the main and interaction effects of psychological control and externalizing behavior were statistically significant for accompanied children, suggesting that externalizing problem behavior moderated the association between maternal psychological control and accompanied children's anxiety (see Table 3). The results of the follow-up simple slope analyses revealed a pattern consistent with an exacerbating process. As indicated in Figure 1, maternal psychological control was positively associated with anxiety for accompanied children with higher levels of externalizing behavior but not for those with lower levels of externalizing behavior. Although the main effects of psychological control and externalizing behavior were statistically significant, the predicted interaction effects failed to reach significance for children with two migrant parents and with only a migrant father, suggesting that externalizing problem behavior did not moderate the association between psychological control and anxiety in left-behind children populations (see Table 3).

**Figure 2:**
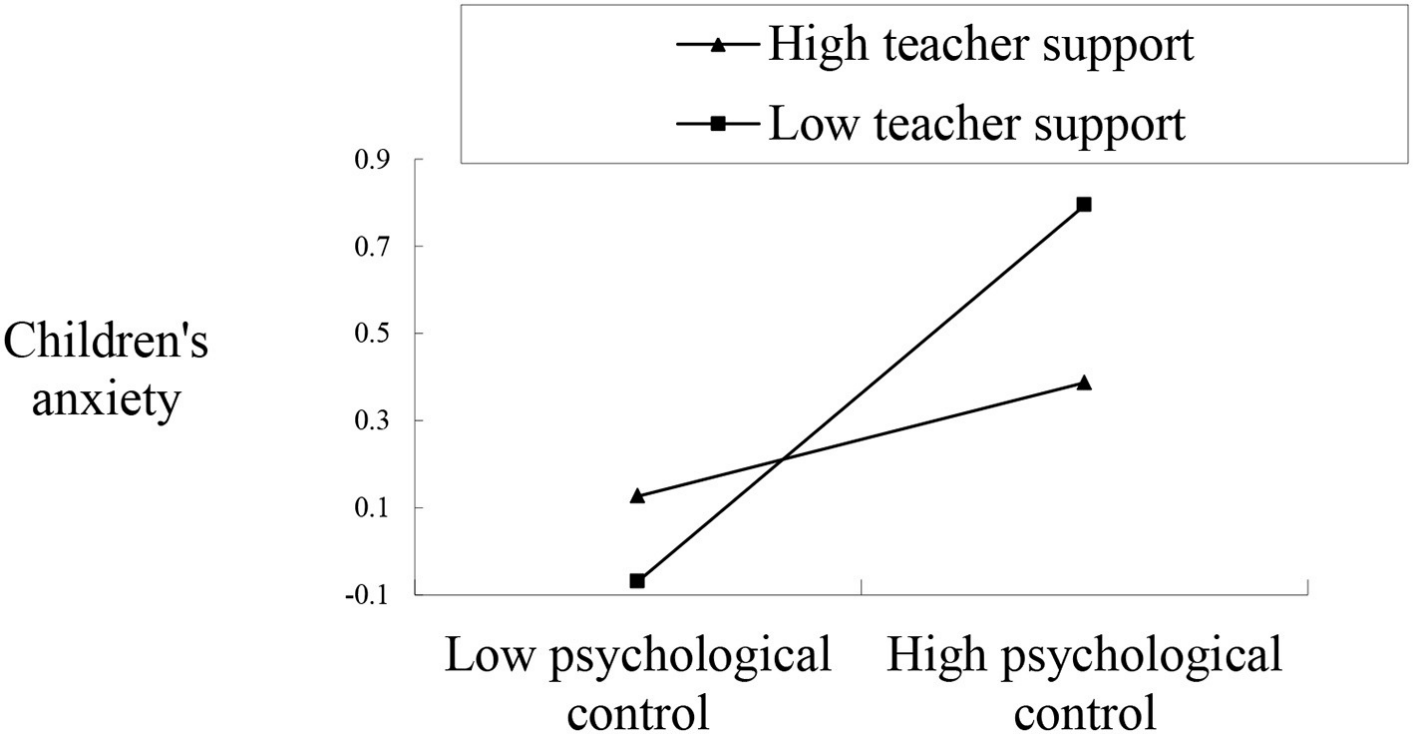
Interaction between maternal psychological control and teacher support in predicting anxiety in children with only a migrant father.

The results from the other three regression models testing the moderating role of teacher support showed that the regression models were statistically significant, explaining 27, 13, and 15% of the variance in anxiety for children with two migrant parents, children with a migrant father, and accompanied children, respectively. Both the main effect of psychological control and its interaction with teacher support were significant for children with a migrant father, suggesting that teacher support moderated the association between psychological control and anxiety in children with a migrant father (see Table 3). The results of the follow-up simple slope analyses demonstrated a pattern consistent with a buffering process. As illustrated in Figure 2, maternal psychological control was positively associated with anxiety for children with a migrant father at lower levels of teacher support, but this association became non-significant for those at higher levels of teacher support. Although the main effects of psychological control and teacher support were statistically significant, the predicted interaction effects failed to reach significance, suggesting that teacher support did not moderate the association between psychological control and anxiety for children with two migrant parents and accompanied children (see Table 3).

## Discussion

Previous research indicates that parental psychological control is associated with children's anxiety (Bebes et al., 2015; Stone et al., 2015; Zhao et al., 2018). The present study extended such findings to left-behind children in rural China. Moreover, this study went beyond prior research by simultaneously examining this association in different families of parents' migrant status and further exploring whether the moderating roles of externalizing problem behavior and teacher support varied as a function of parents' migrant status. The results largely supported our hypotheses, as discussed below.

As anticipated, our analyses of different families of parents' migrant status revealed that children with two migrant parents reported higher levels of anxiety than children with only a migrant father and accompanied children, and no significant difference was found between children with only a migrant father and accompanied children. Attachment theory suggests that loss of the attachment figure (e.g., physical separation) may increase children's vulnerability to anxiety (Brumariu and Kerns, 2010). Thus, this disadvantages experienced by children with two migrant parents may partly be attributable to the lack of secure attachment with their parents. Specifically, although migrant fathers and mothers provide their children with more economic resources, the secure attachment bond between child and parents may be interrupted due to the reduced sensitivity and availability of parental response and the weakened parent–child bonding and communication (Cheng and Sun, 2014), thus contributing to the elevated level of anxiety symptoms. However, children with a migrant father may not only enjoy better financial conditions as a result of their father's earnings but also benefit from their accompanied mother (Zhao et al., 2015), considering that mothers are typically the most dedicated caregivers in the family and that children tend to feel closer to mothers. The company of left-behind mothers is perhaps the primary reason why there was no significant difference in levels of anxiety between children with a migrant father and accompanied children.

By simultaneously investigating the associations between maternal psychological control and children's anxiety in three types of families, the present study found evidence that a higher level of perceived maternal psychological control was associated with more anxiety symptoms in all types of families. This finding is in line with the results of studies across a broad range of samples (Stone et al., 2015; Salaam and Mounts, 2016; Zhao et al., 2018). It has been suggested that parental psychological control thwarts children's autonomy and identity development (Bebes et al., 2015) and is therefore hypothesized to represent a threat to children's emerging sense of self (Stone et al., 2015). Stress-process theory asserts that life events and chronic stress are more likely to result in distress or anxiety when they function to diminish the self (Frazer and Fite, 2016), which may partly explain the negative impact of maternal psychological control on children's anxiety. In addition, the present study also found that children with two migrant parents had a stronger association between maternal psychological control and their anxiety than accompanied children. This finding was basically consistent with our hypothesis and indicated that the association between a negative parenting environment and offspring's maladaptive outcomes was stronger for disadvantaged children with two migrant parents than for accompanied children. This result was also in line with context-dependent theory, which suggests that chronic and cumulative stress may be a source for children's high reactivity to negative environments that lead to their maladaptation (Obradović et al., 2010; Steeger et al., 2017). Specifically, compared with accompanied children, children with two migrant parents may face more challenges and experience more pressure. For instance, they not only suffer from separation from their parents but also experience difficulties in independently solving problems in life and study. Chronic stress may contribute to their emotional arousal, which may make them more sensitive to maternal psychological control. Further research needs to explore the possible mechanism of emotional arousal underlying this association in left-behind children's populations.

This study found that the moderating effect of externalizing problem behavior on the association between maternal psychological control and child anxiety varied as a function of parents' migrant status. Among accompanied children, externalizing problem behavior exacerbated the association between maternal psychological control and their anxiety. This finding was basically in line with those of prior research in intact families (Caron et al., 2006; Pereira et al., 2009; Gere et al., 2012), indicating that psychological control parenting may not be uniquely related to children's anxiety and that the significant association between psychological control and child anxiety may be influenced partly by cooccurring externalizing problem behavior in the same children. Based on this finding, special attention must be paid to helping parents in non-migrant families regulate what their children do, such as guidance, monitoring, and rule setting (Pomerantz and Wang, 2009), to buffer the negative impact of maternal psychological control on their children's anxiety by decreasing the level of children's externalizing problem behavior. However, of note, among two-migrant-parent and only-a-migrant-father children, maternal psychological control was always associated with their anxiety regardless of the level of their externalizing problem behavior. This finding was in line with our anticipation and the viewpoint of context-dependent theory, which suggests that increasing emotional arousal resulting from parental absence may strengthen the negative impact of maternal psychological control on left-behind children's anxiety (Steeger et al., 2017), even though the other risk factors were at a lower level (Wen and Lin, 2012). Based on this finding, it is possible that a fundamental strategy to relieve anxiety or cultivate the well-being of left-behind children was to decrease their parents' migration from rural to urban areas so that more children live in a situation of parental care and nurturance. However, with the rapidly increasing number of children left by both parents or father only in their original rural community, there is still a long way to go in solving the problem of left-behind children's well-being (Luo et al., 2009). Thus, more research is necessary to explore protective buffers against vulnerable situations of parental absence in the future, which may be helpful for developing an effective intervention program to improve the well-being of left-behind children (Zhao et al., 2015).

To better understand the protective factor that benefits left-behind children's adjustment, the present study further explored the moderating role of teacher support in the association between maternal psychological control and children's anxiety in different families of parents' migrant status. It was found that the moderating roles of teacher support varied across parents' migrant status. Among children with only a migrant father, teacher support buffered the impact of maternal psychological control on children's anxiety. This finding was consistent with prior research that demonstrated social support buffering against the vulnerable situations of parental absence (Zhao et al., 2015, 2018). This finding was also consistent with social provisional theory, which suggests that parents and significant others are both important providers of social support and that the relationship with significant others may become increasingly important provisional resources when the parent-child relationship is lacking in social provisions (Furman and Buhrmester, 1985). Thus, it is possible that the teacher becomes an important source of social support serving to protect against anxiety problems for children lacking fathers' care and nurturance. It is noteworthy, however, that the moderating roles of teacher support in the association between psychological control and anxiety were not found among accompanied children and those with two migrant parents, although the main effects of teacher support on the decrease in anxiety were significant in the two groups. Considering that family support matters more than the support of significant others in childhood and adolescence (Runtz and Schallow, 1997), support from fathers may effectively counterweigh the protective role of teacher support in the impact of maternal psychological control on accompanied children's anxiety. In families with two migrant parents, children have the lowest level of family support and are most disadvantaged in terms of anxiety or other health problems; thus, the support of significant others (e.g., teachers) may not sufficiently buffer the negative impact of maternal psychological control on their anxiety. However, special attention is still needed to institutionalize teacher support by training, evaluation, and regulation, given the beneficial roles of teacher support in decreasing the anxiety of two-migrant-parent children and accompanied children and buffering the negative impact of maternal psychological control on the anxiety of only-a migrant-father children. This finding is very encouraging: it is presumably easier to promote the quality of teacher support via school training than to improve the quality of family support (Wen and Lin, 2012), which is hard to influence in migrant families in rural China.

## Limitations

This study suffers from several methodological limitations. First, this study used a cross-sectional design; thus, it is difficult to make causal inferences. Future longitudinal studies need to firmly establish causal associations between variables. Second, the data of this study were solely based on children's reports, which may introduce systematic biases in estimates of associations among the key variables. Third, this study only recruited samples from the Linyi area of China. It remains unknown whether our findings can be generalized to left-behind children in other rural areas of China. Further research needs to recruit representative samples from different regions of rural China to replicate these findings.

## Data Availability Statement

The raw data supporting the conclusions of this article will be made available by the authors, without undue reservation.

## Ethics Statement

The studies involving human participants were reviewed and approved by the Institutional Review Board at the Institute of Psychology, Chinese Academy of Sciences. The participants' legal guardian provided written informed consent to participate in this study.

## Author Contributions

All authors listed in this study made a substantial, direct and intellectual contribution to the work, and approved it for publication. ND: data curation, formal analysis, methodology, and writing—original draft. HB: data curation, formal analysis, and writing—review. JZ: project administration, conceptualization, writing—review and editing, and supervision.

## Conflict of Interest

The authors declare that the research was conducted in the absence of any commercial or financial relationships that could be construed as a potential conflict of interest.
